# Supplementary material for: Exploration of the Misfolding Mechanism of Transthyretin Monomer: Insights from Hybrid-Resolution Simulations and Markov State Model Analysis
Source: Biomolecules. 2019 Dec 17;9(12):889. doi: 10.3390/biom9120889 (PMC6995605; doi:10.3390/biom9120889)
Supplement: Supplementary file 1 [file biomolecules-09-00889-s001.pdf]

Exploration of the Misfolding Mechanism of Transthyretin Monomer: Insights from Hybrid-Resolution Simulations and Markov Model Model Analysis

Shuangyan Zhou[1], Jie Cheng[1], Ting Yang[1], Mingyue Ma[1], Wenying Zhang[1], Shuai Yuan[1, *], Glenn V. Lo[2], Yusheng Dou[2]

[1] Chongqing Key Laboratory on Big Data for Bio Intelligence, Chongqing University of Posts and Telecommunications, Chongqing 400065, China

[2] Department of Chemistry and Physical Sciences, Nicholls State University, P.O. Box 2022, Thibodaux, Louisiana 70310, USA

* Corresponding author

E-mail address: yuanshuai@cqupt.edu.cn

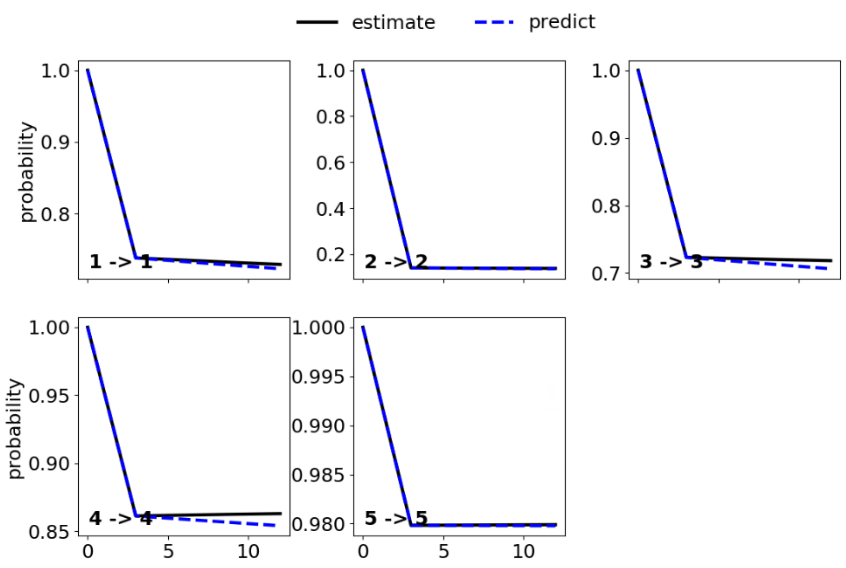

Figure S1. Chapman-Kolmogorov tests of the MSM constructed at lag time of 3 ns with 5 macrostates.

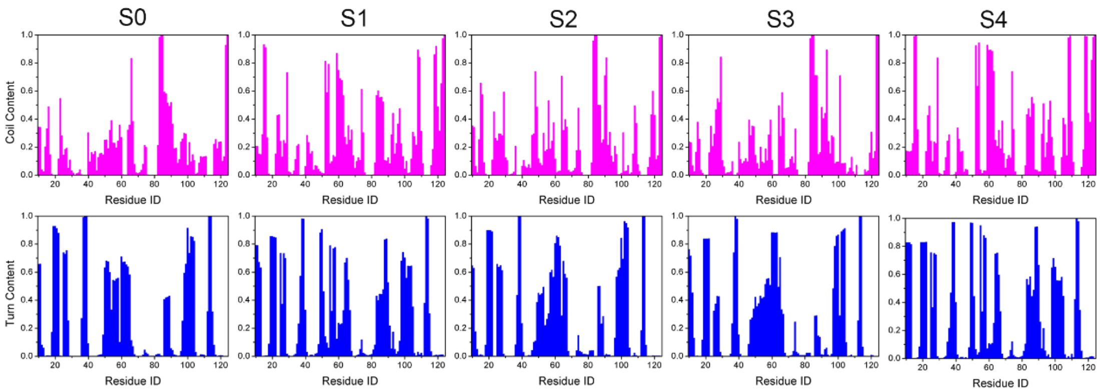

Figure S2. Probabilities of residues in TTR monomer adopting coil and turn structure for each macrostate.

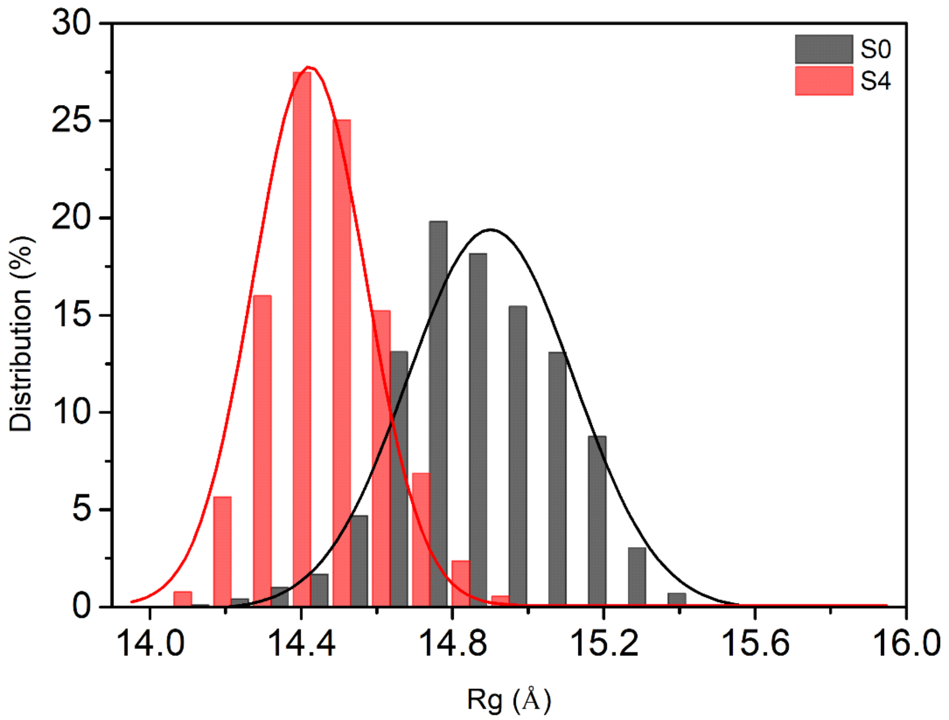

Figure S3. Distribution of radius of gyration (Rg) of macrostates S0 and S4.

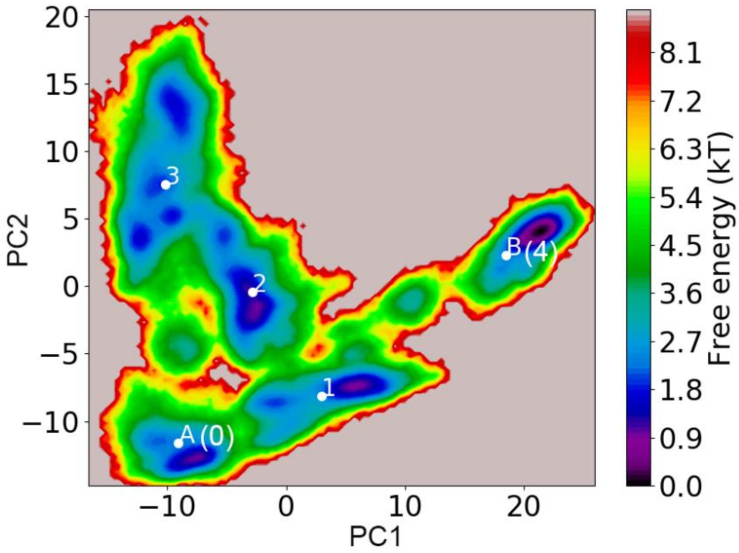

Figure S4. Free energy space of TTR monomer with average location of corresponding macrostates labeled. A and B representing the initial and final states in the transition pathways, respectively.
